# Stakeholder engagement in the development of an upper extremity outcome measure for children with rare musculoskeletal conditions

**DOI:** 10.1186/s40900-023-00479-6

**Published:** 2023-08-08

**Authors:** Caroline Elfassy, Lisa Wagner, Johanne Higgins, Kathleen Montpetit, Laurie Snider, Noémi Dahan-Oliel

**Affiliations:** 1 Faculty of Medicine, School of Physical and Occupational Therapy, McGill University, Montreal, Canada; 2 Clinical Research Department, Shriners Hospital for Children –Canada, 1003 Decarie Boulevard, Montreal, QC H4A 0A9 Canada; 3 Shriners Hospitals for Children, Greenville, USA; 4 Université de Montréal, École de Réadaptation, Montreal, Canada

**Keywords:** Stakeholder engagement, Outcome measure development, Rare musculoskeletal condition, Upper extremity

## Abstract

**Background:**

Upper extremity (UE) involvement is prevalent in 73% of individuals with arthrogryposis multiplex congenita (AMC), yet no AMC-specific outcome measure exists. When developing a measure specific to a population with a rare musculoskeletal condition, clinicians’ and patients’ perspectives and involvement is a crucial and necessary step. This study sought to determine the most clinically useful items for an outcome measure of UE function for children with AMC as defined by caregivers and clinicians.

**Methods:**

To ensure the perspectives and needs of caregivers of children with AMC and clinicians were considered in the development of the UE measure for AMC, a Nominal Group technique (NGT) with caregivers of children with AMC (phase 1) followed by a three-round survey with clinicians (phase 2) were carried out.

**Results:**

Phase 1: Eleven individuals participated in the nominal group technique and identified 32 items. The most important items were Picking up an object (n = 11), Eating (n = 10), Reaching mouth (n = 10), Getting out of bed (n = 10). Phase 2: Invitations to participate to an online survey was sent to 47 experts in the field of AMC, 20 participants completed round 1, 15 completed round 2 and 13 completed round 3. Throughout the survey, participants were asked about movement required to screen the UE, essential domains to be included in the measure, establishing a scoring guide and identifying tasks associated with joint motion and position.

**Conclusion:**

A preliminary version of an UE AMC-specific outcome measure was developed with the help of caregivers’ perspectives and expert opinions.

**Supplementary Information:**

The online version contains supplementary material available at 10.1186/s40900-023-00479-6.

## Abstract (plain language summary)

Arthrogryposis multiplex congenita (AMC) is a rare musculoskeletal condition affects the joints and muscles of the body. In about 70% of the cases, it affects the upper extremities (UE). However, there is no specific outcome measure for UE function in children with AMC. An outcome measure refers to a tool or method used to assess and measure the results or effects of a particular treatment, intervention, or condition. It helps healthcare professionals and researchers understand the impact or outcome of a specific situation, such as the level of improvement or changes in a person's health or function. The goal of this study was to develop such a measure while accounting for the perspectives of youth with AMC, their caregivers and clinicians. To achieve this, a study was conducted in two phases. In the first phase, a Nominal Group technique (NGT) was used to gather input from caregivers of children with AMC. Eleven individuals participated and identified 32 items, with the most important being picking up an object, eating, reaching the mouth, and getting out of bed. In the second phase, a three-round survey was sent to 47 experts in the field of AMC, with 20 participants completing the first round, 15 completing the second round, and 13 completing the third round. The survey asked participants about screening UE movement, essential domains to include in the measure, establishing a scoring guide, and identifying tasks associated with joint motion and position. With the help of caregivers’ perspectives and expert opinions, a preliminary version of an UE AMC-specific outcome measure was developed. This measure will be useful in assessing the UE function in children with AMC and will aid clinicians in developing appropriate treatment plans for this rare condition.

**Supplementary Information:**

The online version contains supplementary material available at 10.1186/s40900-023-00479-6.

## Background

In the last 10 years, patient engagement in health research has emerged as the next evolution in healthcare delivery [1–3]. The Canadian Institute for Health Research’s Strategy for Patient Outcome Research (SPOR) defines patient engagement in health research as “occur[ing] when patients meaningfully and actively collaborate in the governance, priority setting, and conduct of research, as well as in summarizing, distributing, sharing, and applying its resulting knowledge” [4]. Involvement of key stakeholders, which includes patients, in the development of outcome measures to be used in clinical research is essential [5, 6]. Indeed, de Vet et al. (2011) state that instruments to measure functioning should be developed in close cooperation with experts (i.e. clinicians who have extensive expertise on target population or patients’ lived experience) [7]. When developing a measure specific to a population with a rare musculoskeletal (MSK) condition, clinicians’, patients’ and caregivers’ perspectives and involvement is a crucial and necessary step. One such MSK condition is arthrogryposis multiplex congenita (AMC).

AMC is a term used to describe a group of congenital conditions characterized by joint contractures in two or more body areas [8]. Treatment involves rehabilitation and orthopedic surgery with multidisciplinary team members to maximize the capacity and performance of the child with AMC to provide the best outcomes throughout the lifespan [9]. The multidisciplinary team consists of the child and their family with varied healthcare professionals (e.g., orthopedics, genetics, neurology, rehabilitation) depending on the child’s involvement. AMC can affect the upper and/or lower extremities as well as other body areas such as the gastrointestinal, genitourinary and central nervous systems [10]. The lower extremities are involved in 89–95% of cases [11], while individuals have upper extremity (UE) involvement in about 73% of cases [12]. The most frequent clinical presentation of the UE at birth has been described as internal rotation of the shoulders, extension of the elbows, flexion of the wrists, thumb-in-palm deformity, and variable movement in the fingers [12, 13]. According to Hamdy et al. (2019), UE function is the most determining aspect of quality of life and independent living for individuals living with AMC as it pertains to daily activities such as dressing, perineal hygiene, grasping, use of mobility aids when needed, and feeding [11].

According to Wagner et al. [9], rehabilitation practitioners (i.e., occupational therapists (OTs) and physical therapists (PTs)) enable function and help individuals with AMC participate in meaningful activities [14–16]. Outcome measures can be defined as a tool or method used to assess and measure the results or effects of a particular treatment, intervention, or condition. The use of outcome measures can help clinicians evaluate a child’s capacity and performance and determine a treatment plan based on the child’s needs. OTs and PTs use a variety of outcome measurement and evaluation tools to assess a patient’s level of functioning. Although many outcome measures exist, some were developed specifically to assess and evaluate a region of the body and others were tailored for specific pediatric populations [17].

Currently, clinicians utilize generic measures aimed at overall assessment of functional performance and/or motor function as there are no AMC-specific outcome measures that exist. These standardized measures provide important information on the child’s level of function. However, they were not developed for children with an UE deformity, do not inform as to which specific joint or muscle limits functional performance, and may not reflect the adaptations or compensatory strategies used by children with AMC (for example, using feet or mouth for activities). For example, the Functional Dexterity Test was developed to measure manual dexterity skills for functional fine motor coordination tasks performance in adults and children [18] whereas the Shriners Hospital Upper Extremity Evaluation (SHUEE) was designed to evaluate UE function in hemiplegic cerebral palsy in children between 3 and 18 years of age. Although some generic UE outcome measures have been used with the AMC population, these have not been validated for a standardized evaluation [19]. Indeed, during the Second International Symposium on Arthrogryposis held in St. Petersburg, Russia in 2014, a need for the development of a standardized assessment of short- and long-term outcomes was identified [20]. The authors explored what is currently known regarding participation among children and youth with AMC [21] as well as identified the needs surrounding rehabilitation according to youth with AMC and caregivers [22]. In a preliminary study on the development of a standardized AMC-specific outcome measure, the authors identified an item bank of the most frequently reported pediatric performance-based outcome measure (PBOM) of UE function and linking their content to the International Classification of Functioning, Disability and Health (ICF) [23]. In the scoping review, the authors defined UE function as UE coordination, motor function, sensation, muscle strength, and stabilization. In order to engage caregivers and clinicians, the aim of this study consisted of identifying, according to their expertise, the most clinically useful items for an outcome measure of UE function for children with AMC. The ICF framework and definitions were used exclusively for all phases of the development of the UE AMC-specific outcome measure.

## Methods

This study received institutional approval in May 2021 (CAN2103) and ethics approval from the institutional review board of McGill University’s Faculty of Medicine in May 2021 (A03-B15-21A).

To ensure the perspectives and needs of caregivers of children with AMC as well as clinicians were considered in the development of the UE measure for AMC, a Nominal Group technique (NGT) with caregivers of children with AMC (phase 1) followed by a three-round survey with clinicians (phase 2) were carried out. These two phases are described below.

### Phase 1: patient engagement nominal group technique

The NGT is a structured face-to-face small group discussion aimed at reaching consensus and providing a prompt result for researchers [24, 25]. The NGT gathers information by asking individuals to respond to questions posed by a moderators, and then asking participants to prioritize the ideas or suggestions of all group members [24]. The four steps used for the NGT included: (1) generating items, (2) recording items, (3) discussing items, and (4) voting on items [24–26]. During a breakout session at the 14th Annual Arthrogryposis Multiplex Congenita Supper Inc. (AMCSI) Conference in July 2019 in Norfolk, USA, youth and adults with AMC and their caregivers were invited to participate in an open group discussion to gather and exchange ideas regarding the future development of an UE outcome measure specific for children with AMC. As AMC is comprised of a group of heterogeneous conditions with varying levels of severity and involvement, any individual presenting with multiple congenital contractures as well as other comorbidities such as CNS involvement and their caregivers were invited to participate. The session was audio-recorded, and interested participants were asked to verbally consent to participate.

The meeting took place in a closed room where all participants were able to talk freely and confidentially. An opening statement where an agenda as well as the importance of each member’s contribution was presented by PowerPoint. In order to address the first step of the NGT, the overall statement presented looked to answer the following:

When a therapist or rehabilitation professional is assessing you or your child’s arm/shoulder/elbow/wrist/hand/finger, what do you think is an important aspect or item to consider in this evaluation (it could be a simple task or an activity that requires many steps.

Each participant was asked to answer the above statement, with as many responses, silently and independently, on a piece of paper provided by the research team. The second step consisted of engaging all participants, one at a time, in a round-robin feedback session to concisely record each item (without debate). The round-robin continued until each participants’ items had been documented. The third step entailed discussing each item to determine the clarity and importance. For each item, the principal author asked participants if they had any questions or comments regarding the item or if they required clarification. Finally, the fourth step involved voting on the items generated. The voting consisted of asking each participant to categorize each of the identified items as A) Important and essential, B) Important but not essential, and C) not relevant. Each participant voted independently.

### Phase 2: clinician opinion using a three-round survey

**Fig. 1:**
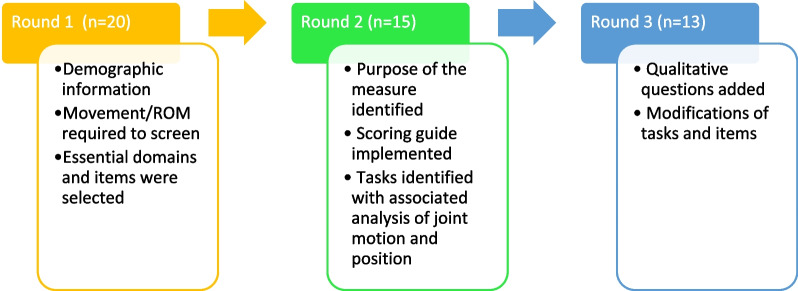
Description of each survey round for the clinicians

The survey consisted of pre-selected items drawn from preliminary work [21–23] as well as the findings of the NGT. The participants included in the survey were clinicians as phase 2 focused not only on the development of items but also on creating a scoring guide to be used during clinical evaluations. Approximately 50 clinicians in the field of AMC were invited to participate in a three-round survey. These clinicians included PTs, OTs, certified hand therapists, orthopedic surgeons, and physical rehabilitation technicians. The clinicians were identified during the 3^rd^ International Arthrogryposis Symposium in Philadelphia, USA, in 2018. Invitations to participate in the survey process were sent by e-mail, explaining the purpose of the project, with a link to an electronic survey using the Qualtrics online platform. Participants were asked to complete the survey within a 1-month timeframe. A weekly reminder was sent using the Qualtrics software. Clinicians were eligible to participate regardless of location or setting of practice, as long as they had at least 2 years’ experience working with the pediatric AMC population. A summary of the surveys and can be found in Fig. 1.

Based on findings of preliminary work [21–23], the authors prepared a draft version of the survey which included the purpose of the measure, the inclusion/exclusion criteria, consent waiver link, demographic information, as well as “must have” items of the outcome measure to be included.

The first-round of the survey asked each clinician a series of demographic questions (i.e., profession, country of residence, type of work setting, years of practice, years of experience with AMC, number of children with AMC they have worked with, healthcare professionals on their team). In addition, clinicians were presented a series of questions related to domains of performance-based items and domains of scoring such as range of motion. Similarly to Lawshe’s method of content validation, expert clinicians were asked to rate each item as essential, useful but not essential, and not necessary [27]. A comment box was made available throughout the survey and for each item should the participants have additional comments to raise. Invitations to participate in the second and third round survey were only sent to clinicians who had previously completed or partially completed the prior rounds.

During the second-round survey, clinicians were presented with the preliminary version of the purpose of the measure and the scoring guide. Clinicians were asked to determine item clarity and word choice for the different subtasks. They were also asked which joints should be the focus as well as the hierarchy of scoring used for analysis of joint motion and position score.

For the third and final round, the clinicians were presented with the final version of the purpose of the measure and the scoring guide. Clinicians were asked to look at the measure as a whole and provide any comments regarding scoring, overall layout and clarity of content.

Descriptive analysis was provided for both phases, particularly the three-round surveys. Results arising from partially completed surveys we’re not included and were considered as non-complete for our analysis.

## Results

### Phase 1: patient engagement nominal group technique

During the 14^th^ Annual Arthrogryposis Multiplex Congenita Support Inc. (AMCSI) Conference, a total of 11 individuals (7 mothers, 3 fathers, and 1 adult with AMC) participated in the NGT and identified 32 items. The detailed list of identified items can be found in Table 1. According to the voting system, the most important and essential items identified were the following: Picking up an object (n = 11), Eating (n = 10), Reaching mouth (n = 10), Getting out of bed (n = 10), Using spoon/fork (n = 9), Putting on pants (n = 9), Dressing (donning and doffing coat) (n = 9), Brushing teeth (n = 9), and Ability to participate in social games with family members (n = 9).

**Table 1:** Items pool during the patient engagement nominal group technique

Item #	Item name	Voting score
1	Ability to participate in social games with family members (board games, ball catch)	9
2	Ability to participate in recess at school with peers (elementary)	7
3	Ability to use adapted objects	8
4	Autonomy (individualized goals)	10
5	Putting on pants	9
6	Satisfaction with completion of tasks	7
7	Eating (holding a bottle)	10
8	Using spoon/fork	9
9	Range of motion of shoulder, elbow, wrist, and fingers	7
10	Picking up an object	11
11	Constantly re-evaluating	7
12	Everyday living	9
13	Milestones-appropriate goals	9
14	Holistic, global approach	6
15	Writing	8
16	Bilateral hand use	8
17	Active vs. passive ROM	8
18	Reaching mouth	10
19	Playing at midline	8
20	Throwing	7
21	Dressing (donning and doffing coat)	9
22	Brushing teeth	9
23	Getting out of bed	10
24	Natural vs. clinical environment	7
25	Completing activities per age (developmental milestones)	8
26	Routine breakdown	8
27	Preparing food	7
28	Driving a car	7
29	Opening a door using a key	7
30	Managing hair	8
31	Light switches	8
32	Scoring and recommendations (caregivers wanted to understand what the scores of their child’s performance on outcome measures represented and how it impacted treatment recommendations)	7

### Phase 2: clinician opinion using a three-round survey

Of the 47 clinicians invited to the first-round of survey, 20 completed the first round, 15 the second round, and 13 completed the third round. Demographic information of the participants for each completed survey round can be found in Table 2.

**Table 2:** Demographic information of the clinicians in the Three-Round Survey

Round 1 (n = 20)			Round 2 (n = 15)			Round 3 (n = 13)		
Occupation	Country	Work setting	Occupation	Country	Work setting	Occupation	Country	Work setting
Orthopedic surgeon (n = 9)	USA (n = 13)	Hospital (n = 16)	Orthopedic Surgeon (n = 6)	USA (n = 10)	Hospital (n = 12)	Orthopedic surgeon (n = 4)	USA (n = 8)	Hospital (n = 11)
PT (n = 4)	Canada (n = 3)	University (n = 1)	PT (n = 2)*	Canada (n = 2)	Speciality Hospital (n = 2)	PT (n = 4)*	Canada (n = 2)	University (n = 1)
OT (n = 6)	Poland (n = 2)	Speciality Hospital (n = 2)	OT (n = 7)*	Poland (n = 1)	Rare Disorder Center (n = 1)	OT (n = 5)*	Poland (n = 1)	Rare Disorder Center (n = 1)
CHT (n = 1)	Sweden (n = 1)	Rare Disorder Center (n = 1)		Sweden (n = 1)			Sweden (n = 1)	
	Norway (n = 1)			Norway (n = 1)			Norway (n = 1)	

OT = occupational therapist, PT = physical therapist, CHT = certified hand therapist

^*^Value may change from round to round as invitations to participate in subsequent rounds were sent to clinicians that had also partially completed the preceding survey

The first-round survey questionnaire can be found in Additional file 1. The clinicians (n = 20) had on average 17.86 (2–35 years) years of experience working with children and youth with AMC. They oftentimes worked with a multidisciplinary team consisting of pediatricians (n = 5), orthopedic surgeons specializing in different limbs (n = 19), nurses (n = 15), orthotists (n = 17), OTs (n = 17), PTs (n = 20), physiatrists (n = 3), geneticists (n = 10), neurologists (n = 6), social workers (n = 13), psychologists (n = 5), speech language pathologists (n = 4), and neurosurgeons (n = 2). The most important movements for UE screening according to the clinicians were elbow flexion, forearm supination, wrist extension, and finger flexion. Other important UE screening movements can be found in Table 3. Additionally, based on the results of the first-round survey, the domains deemed to be essential to be evaluated in the UE outcome measured included UE weight bearing, UE to change body position, grasp, release, reach, fine hand manipulation, dressing, feeding, and toileting. The Throwing and Catching domains were deemed useful but not essential by the clinicians. Specific items can be found in Table 4.

**Table 3:** Most important movement for the upper extremity screening rated by 20 clinicians (Round 1 Survey)

Shoulder	Elbow	Forearm	Wrist	Fingers	Thumb
Flexion (n = 19)	Flexion (n = 20)	Supination (n = 20)	Extension (n = 20)	Flexion (n = 20)	Opposition (n = 18)
External rotation (n = 19)	Extension (n = 15)	Pronation (n = 16)	Flexion (n = 17)	Extension (n = 17)	Extension (n = 16)
Abduction (n = 17)					Flexion (n = 15)

**Table 4:** Results of essential domains and items rated by 20 clinicians (Round 1 Survey)

Domains	Essential items
UE weight bearing	Uses non-dominant hand to stabilize self (n = 10)
	Uses upper extremity to weight bear in prone (n = 9)
	Uses upper extremity to weight bear in sitting with hands forward (n = 9)
Using the UE to change body position	Pushes self from supine to sitting (n = 18)
	Using UE to use mobility aid (n = 16)
	Pushes self from sitting to standing (n = 14)
	Transferring from one body position to another (n = 14)
Grasp	Picking up a medium object (shoes, water bottle, plate etc.) (n = 20)
	Picking up a small object (coin, bead, pencil, etc.) (n = 17)
Release	Releasing a medium object (shoes, water bottle, plate, etc.) (n = 17)
Reach	Reaching for an item from waist level (n = 16)
	Reaching for an item by crossing the midline (n = 12)
Fine hand manipulation	Write your name or draw something (n = 20)
	Opening a jar (n = 15)
	Closing a jar (n = 12)
Dressing	Puts on clothes over-head (shirt, sweater, hat) (n = 20)
	Pulls on pants (n = 20)
	Pulls down (remove) pants (n = 20)
	Removes shirt (n = 19)
	Puts on shoes (n = 17)
	Puts on open shirt (n = 16)
	Pulls up zipper (n = 15)
	Starts a zipper (n- = 14)
	Puts on socks (n = 14)
	Removes shoes (n = 14)
	Buttoning (n = 13)
	Unbuttoning (n = 13)
Feeding	Reaches mouth (n = 20)
	Picks up food using fork (n = 17)
Toileting	Places sticker on lower back (proxy for reaching to wipe buttocks) (n = 19)

Based on the results from the first round, the preliminary version of the UE outcome measure was created (Additional file 2). In this second survey round, the purpose of the measure was revised (i.e. describe impairments, activity limitations, and participation restrictions in the performance of daily tasks in children with AMC to guide treatment decision-making and evaluation of treatment effectiveness for the UE), a scoring guide for task completion was developed (Table 5), 12 tasks and 3 descriptive questions (Table 6) were generated, and a scoring table including analysis of joint motion and position was derived. Fifteen clinicians completed the survey, three partially completed it, and two did not respond. Clinicians in the second-round survey were asked about terminology and which joints to analyse for each task. Table 7 presents the different terminology choices offered to the clinicians. Based on the scoring provided by the clinicians, additional joints such as wrist in task 2, forearm in task 4, thumb, fingers, and wrist in task 6 and 7, fingers, wrist, forearm, and shoulder in task 10 and shoulder in task 12 were added to be analyzed in the scoring sheet.

**Table 5:** Scoring guide for task completion (Round 2 Survey)

Score	Description	Example
0	Unable	The child is unable to complete any component of the task
1	Partial completion of task passively	The child can partially complete the task using passive range of motion
2	Partial completion of task actively	The child can partially complete the task using active range of motion
3	Completion of task passively	The child can complete the task using passively range of motion
4	Completion of task actively	The child can complete the task using active range of motion

**Table 6:** Descriptive questions developed in Round 2 Survey

1. Does the child use their arms for using a mobility device?
2. Does the child use their arms for shifting/changing/moving body (getting on or off couch/toilet etc.)?
3. Does the child use a splint for the tasks included?

**Table 7:** Task terminology choice and rating (n = 15) (Round 2 Survey)

Task	Terminology choice
1. Grasp/pick up a cheerio, bring it to your mouth, place it back down in front of you, and release it/let it go	Pick up (n = 14)
	Let it go (n = 9)
2. Grasp/pick up a water bottle/can, bring it to your mouth, place it back down in front of you, and release it/let it go	Pick up (n = 9)
	Water bottle (n = 13)
	Let it go (n = 9)
3. Open the jar, pour out a few beads/macaroni/buttons, string 3 together, and close the jar	
	Beads (n = 13)
4. Pick up the crayon/marker, write your name on this piece of paper, fold the paper, and cut it using the scissors	
	Marker (n = 12)
5. Pick up the Play-Doh using the fork and bring it to your mouth	N/A
6. Reach for a small-size ball (e.g., tennis ball) placed on the floor, throw the ball underhand. Repeat task, throwing ball overhead	
	N/A
7. Reach for a medium-sized ball (e.g., basketball) placed on the floor, throw the ball underhand. Repeat task, throwing ball overhead	
	N/A
8. Put on a T-shirt overhead and take off the T-shirt	
	N/A
9. Put on vest/sweater with zipper, fasten the zipper, pull it all the way up, and pull it back down	Vest (n = 9)
10. Pull down your pants, reach bum/buttocks area, place a sticker on bum/buttocks area [proxy for wiping after bowel movement], place sticker in between legs [proxy for wiping after urination], and pull pants back up	
	Buttock (n = 9)
	Buttock (n = 9)
11. Put on a sock and take it off	N/A
12. Show us how you move from lying down on your back to a sitting position	
	N/A

N/A signifies there was no preferred word choice option for that particular task

The third and final round survey was sent to 18 participants (Additional file 3), 13 participants completed the survey in its entirety and 5 partially completed it. Modifications to the UE outcome measure included substituting the arc of motion of a joint to a specific direction of movement (i.e. no external rotation, partial external rotation, full external rotation vs. internal rotation, neutral, external rotation), modifying the hierarchy of scoring, and adding a scoring row to reflect a specific item (i.e. reveal the bimanual nature of the task (e.g. stabilizing with one hand and fold/cut with another). Task 12 was removed as it was replaced with a table consisting of various transfers capacities that the child can perform (i.e. bed positioning, lying to sitting, sitting to standing, toilet transfer, bathtub/shower transfer, getting in and out of a car) in the descriptive questions section (Table 8). Task 12 was no longer specific to only transferring from lying to sitting but rather assessing all transfers required in daily routines. The qualitative questions were edited to include a propelling a manual wheelchair and operating a motorized wheelchair.

**Table 8:**
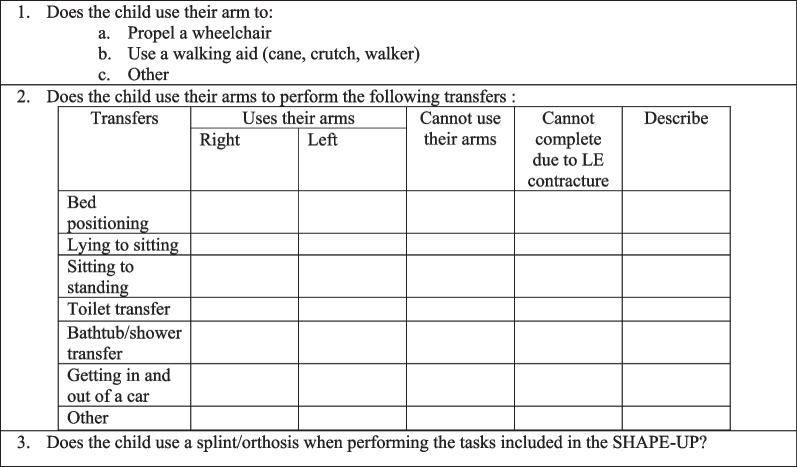
Descriptive questions in Round 3 Survey

## Discussion

The goal of this study was to describe the process of developing a new outcome measure of UE function for children with AMC and identify the most clinically useful items to be included as determined by individuals with AMC and their caregivers, and clinicians. The results from the NGT and the survey yielded a first of its kind preliminary version of an AMC-specific UE outcome measure. The outcome measure includes 11 tasks with a total of 47 subtasks and 3 qualitative descriptive questions. A complete scoring guide as well as an analysis of joint motion and position was developed.

According to the findings in phases 1 and 2, the items identified were predominantly related to the Body Functions and Structures and the Activity domains of the ICF. This result is not entirely surprising as specific outcome measures related to other pediatric conditions have shown the same results. For example, in a previously published systematic review, the authors aimed to define and link the meaningful concepts of items contained in three commonly administered standardised UE outcome measures (i.e. Melbourne Assessment, Quality of Upper extremity Skills Test (QUEST), Assisting Hand Assessment (AHA)) used in cerebral palsy [28]. According to the review, the Melbourne Assessment was reported as a measure of the Activity domain of the ICF, the QUEST was related to both the Body functions and Activity domains of the ICF, and the AHA was consistent with the Activity domain of the ICF [28]. Additionally, an article showcasing the overview of assessments and classification tools used to understand and measure UE function associated with children with spasticity indicated that the Kids-Assisting Hand Assessment, Mini-Assisting Hand Assessment, Children’s Hand-use Evaluation Questionnaire, ABILHAND-Kids, Canadian Occupational Performance Measure, and Goal Attainment Scaling were linked to the Activity domain of the ICF, the Pediatric Motor Activity Log was related to Body Function and Structure domain of the ICF, and the Melbourne Assessment, QUEST, Box and Block of Manual Dexterity, SHUEE were related to Body Function and Structure and Activity domains of the ICF [29]. Pediatric specific UE outcome measures used in clinical settings across different diagnoses have been shown to be focused more on the Body Functions and Structures and Activity Domains of the ICF. Indeed, there was a need surrounding the Participation and Environment domain of the ICF that was identified in a recent publication [22]. Youth with AMC stated that rehabilitation focused primarily on physical limitations which did not always correspond to the youth’s specific participation needs [22]. Therefore, the throwing and catching subtasks were kept in the measure even though they were deemed useful but not essential in the survey, as it was important to consider participation based on the needs identified by key stakeholders, in this case individuals with AMC.

The clinical implications of involving both individuals with lived experience as well as clinicians with expertise in the field of AMC in the development of an outcome measure is twofold. The first implication is having a complete picture of the needs surrounding the evaluation and intervention provided to the child with AMC. Phase 1 of this project (i.e. NGT) allowed for patient engagement and highlighted the importance of including the Participation domain of the ICF in the development of UE outcome measure. Involving youth and caregivers in the development of outcomes and clinical research has been shown to be feasible and valuable to studies [30]. The second clinical implication is to help increase awareness and public interest of children and youth with rare musculoskeletal conditions. Establishing a network of patient partners and clinicians in rare diseases is important to help increase the quality of studies being published and disseminating the results to the population in question.

### Limitation and future work

Although youth with AMC and caregivers were invited to participate in the NGT at the 14th Annual AMCSI Conference, only the caregivers attended the breakout session. However, findings from previously published studies regarding the needs of youth with AMC [21, 22] were included in the development of the preliminary version of the outcome measure.

Due to the scope of study, initial validation of the measure could not be completed at this current time. While this is the first step towards finalizing a pediatric AMC-specific UE outcome measure, further assessment of reliability, construct validity and responsiveness is required and is planned with the next phase of this project. The development of a psychometrically sound UE measure in AMC will elevate current practice and assist in establishing the effectiveness of surgical and non-surgical therapies.

## Conclusion

A preliminary version of an UE AMC-specific pediatric outcome measure was developed with the contribution of patient engagement and clinicians’ opinions consisting of 11 task and 3 descriptive qualitative questions. The measure includes a scoring guide for task completion as well as a joint motion and analysis section to determine which joints of the UE are limiting the child’s with AMC capacity to complete the task.

### Supplementary Information

**Additional file 1.** First-round survey questionnaire.

**Additional file 2.** Second-round survey questionnaire.

**Additional file 3.** Third-round survey questionnaire.

## Data Availability

The datasets used and/or analysed during the current study are available from the corresponding author on reasonable request.

## Abbreviations

AMC: Arthrogryposis multiplex congenita
NGT: Nominal group technique
ICF: International classification of function, disability and health
UE: Upper extremity
OT: Occupational therapy
PT: Physical therapy
SHUEE: Shriners hospital upper extremity evaluation
QUEST: Quality of upper extremity skills test
AHA: Assisting Hand assessment
