# Supplementary material for: Stakeholder engagement in the development of an upper extremity outcome measure for children with rare musculoskeletal conditions
Source: Res Involv Engagem. 2023 Aug 8;9:64. doi: 10.1186/s40900-023-00479-6 (PMC10408044; doi:10.1186/s40900-023-00479-6)
Supplement: Supplementary file 1 — Additional file 1. First-round survey questionnaire. [file 40900_2023_479_MOESM1_ESM.pdf]

Good day,

Our research team at the Shriners Hospital for Children - Canada is working on a project titled: **Development of the Shriners Hospitals Arthrogryposis Pediatric Evaluation - UPper Extremity (SHAPE-UP)**.
The purpose of the SHAPE-UP is to assess UE function in children with AMC with upper limb involvement. The SHAPE-UP will describe the impairments, activity limitations, and participation restrictions in the performance of daily tasks in children with AMC to guide treatment decision-making and evaluate treatment effectiveness for the UE.

We are inviting you to complete a survey that will help us gather information on test items specific to the UE. We will ask you some questions about your professional background and experience, as well as questions specific to test items when assessing children and youth with AMC. The survey takes approximately 20-30 minutes to complete.

By completing this survey, you consent to participate in the study. Please refer to the attached PDF for full consent discussion and details.

We thank you in advance for your participation and contribution to this project.

Please feel free to forward this questionnaire to other clinicians (occupational and physical therapists, physical rehabilitation technicians, physicians, and certified hand therapists) with 2 years or more of experience working with children and youth with AMC. Your help and support is very much appreciated.

If you have any questions regarding the survey, please contact the clinical research coordinator Kathleen Montpetit at kmontpetit@shrinenet.org

SHAPE-UP Purpose and Consent

Development of the Shriners Hospitals Arthrogryposis Pediatric Evaluation - UPper Extremity (SHAPE-UP)

You are being asked to take part in a research study because you are a clinician involved in the care of children with arthrogryposis multiplex congenita (AMC) with at least 2 years experience.

A major component in this process is to include the expertise of experienced practitioners worldwide, such as you. This survey will help us gather information about what are the most clinically important items to consider when evaluating the upper extremity (UE) function of children with AMC.

The purpose of the SHAPE-UP is to assess UE function in children with AMC with upper limb involvement. The SHAPE-UP will describe the impairments, activity limitations, and participation restrictions in the performance of daily tasks in children with AMC to guide treatment decision-making and evaluate treatment effectiveness for the UE.

Please read the consent form attached below. By proceeding with the survey, you acknowledge having read and understood the consent discussion and consent to participate in the study.

Click here: SHAPE-UP Consent Form

Demographic Information

What is your occupation?

- ○ Occupational Therapist
- ○ Physical Therapist
- ○ Physician
- ○ Certified Hand Therapist
- ○ Physical Rehabilitation Technician (TRP)
- ○ ________ Other (Please specify)

What specialty of medicine do you practice?

____

In what country do you currently work?

____

In which type of setting are you currently working?

- ☐ Hospital
- ☐ Community Center
- ☐ Rehabilitation Center
- ☐ Private Center/Private Practice
- ☐ School-Based Practice
- ☐ ________ Other (Please Specify)

Do you have more than 2 years experience working with children/youth (1-21 years) with AMC?

- ○ Yes
- ○ No

How many years have you been working with children/youth (1-21 years) with AMC?

____

How many children/youth with AMC have you worked with?

[ ]

Which healthcare professionals do you work with when caring for children with AMC? Choose all that apply:

- ☐ Pediatrician
- ☐ Orthopedic Surgeon
- ☐ Nurse
- ☐ Orthotist
- ☐ Occupational Therapist
- ☐ Physical Therapist
- ☐ Physiatrist
- ☐ Geneticist
- ☐ Neurologist
- ☐ Social Worker
- ☐ Psychologist
- ☐ Speech Language Pathologist
- ☐ [ ] Other (Please Specify)

Elements of the SHAPE-UP: ROM

Which movement of the **SHOULDER** do you feel is necessary to screen for UE function? (Choose all that apply)

- ☐ ADduction
- ☐ ABduction
- ☐ Flexion
- ☐ Extension
- ☐ Internal rotation
- ☐ External rotation
- ☐ [ ] Other (Please specify)

Which movement of the **ELBOW** do you feel is necessary to screen for UE function? (Choose all that apply)

- ☐ Extension
- ☐ Flexion

Which movement of the **FOREARM** do you feel is necessary to screen for UE function? (Choose all that apply)

- ☐ Pronation
- ☐ Supination

Which movement of the **WRIST** do you feel is necessary to screen for UE function? (Choose all that apply)

- ☐ Flexion
- ☐ Extension
- ☐ Ulnar deviation
- ☐ Radial deviation

Which movement of the **FINGERS** do you feel is necessary to screen for UE function? (Choose all that apply)

- ☐ Flexion
- ☐ Extension
- ☐ ABduction

☐ ADduction
☐ ______ Other (Please specify)

Which movement of the **THUMB** do you feel is necessary to screen for UE function? (Choose all that apply)

☐ Flexion
☐ Extension
☐ ADduction
☐ ABduction
☐ Opposition

Elements of the SHAPE-UP: Scoring

How should the items of the SHAPE-UP be scored? Please rank your response by dragging and dropping in the order your prefer.

Timed

Level of assistance
0 – Unable to complete
1 – Able to complete with human assistance
2 – Able to complete with adaptive equipment
3 – Able to complete independently

Level of performance (ability to complete the task)

Qualifying the position of the limb segments for the specific item (e.g. positional analysis in the SHUEE)

Other (Please specify) ______

Please provide any additional comments regarding scoring options.

______

Elements of the SHAPE-UP: Items

For the Item Block of this questionnaire, please keep in mind that the following items are **performance-based**. This means that we want the child with AMC to perform these items in the setting while the clinician observes them complete the task.

The items fall under 11 subdomains. These domains are:
- Upper Extremity Weight Bearing
- Using the Upper Extremity to change Body Position
- Grasp
- Release
- Reach
- Fine Hand Manipulation
- Throwing
- Catching
- Dressing
- Feeding
- Toileting

For the domain **"Upper Extremity Weight Bearing"** please rate the following items for inclusion in the SHAPE-UP measure as well as add any additional comments. These could include changing the wording, adding a missing item, or changing the meaning of the item.

	Essential	Useful but not essential	Not necessary	Comment
Uses non-dominant hand to stabilize self	☐	☐	☐	☐ ______

	Essential	Useful but not essential	Not necessary	Comment
Uses upper extremity to weight bear in prone	☐	☐	☐	☐
Uses upper extremity to weight bear in sitting with hands forward	☐	☐	☐	☐
Uses upper extremity to weight bear in sitting with hands to the side	☐	☐	☐	☐
Uses upper extremity to weight bear in sitting with hands behind body	☐	☐	☐	☐

Please provide any additional comments for the domain **"Upper Extremity Weight Bearing"**

For the domain **"Using the Upper Extremity to Change Body Position"** please rate the following items for inclusion in the SHAPE-UP measure as well as add any additional comments. These could include changing the wording, adding a missing item, or changing the meaning of the item.

	Essential	Useful but not essential	Not necessary	Comment
Get down and crawl towards me	☐	☐	☐	☐
Pushes self from supine to sitting	☐	☐	☐	☐
Pushes self from sitting to standing	☐	☐	☐	☐
Transferring from one body position to another	☐	☐	☐	☐
Pushes self using upper extremity (bum scoot)	☐	☐	☐	☐
Using upper extremity to use mobility aid	☐	☐	☐	☐

Please provide any additional comments for the domain **"Using the Upper Extremity to Change Body Position"**

For the domain **"Grasp"** please rate the following items for inclusion in the SHAPE-UP measure as well as add any additional comments. These could include changing the wording, adding a missing item, or changing the meaning of the item.

	Essential	Useful but not essential	Not necessary	Comment
Picking up a large object (box, backpack, jacket, etc.)	☐	☐	☐	☐
Picking up a medium object (shoes, water bottle, plate, etc.)	☐	☐	☐	☐
Picking up a small object (coin, bead, pencil, etc.)	☐	☐	☐	☐

Please provide any additional comments for the domain **"Grasp"**

For the domain **"Release"** please rate the following items for inclusion in the SHAPE-UP measure as well as add any additional comments. These could include changing the wording, adding a missing item, or changing the meaning of the item.

	Essential	Useful but not essential	Not necessary	Comment
Releasing a large object (box, backpack, jacket, etc.)	☐	☐	☐	☐
Releasing a medium object (shoes, water bottle, plate, etc.)	☐	☐	☐	☐
Releasing a small object (coin, bead, pencil, etc.)	☐	☐	☐	☐

Please provide any additional comments for the domain **"Release"**

For the domain **"Reach"** please rate the following items for inclusion in the SHAPE-UP measure as well as add any additional comments. These could include changing the wording, adding a missing item, or changing the meaning of the item.

	Essential	Useful but not essential	Not necessary	Comment
Reaching for an item from floor level	☐	☐	☐	☐
Reaching for an item from waist level	☐	☐	☐	☐
Reaching for an item overhead	☐	☐	☐	☐
Reaching for an item by crossing the midline	☐	☐	☐	☐

Please provide any additional comments for the domain **"Reach"**

For the domain **"Fine Hand Manipulation"** please rate the following items for inclusion in the SHAPE-UP measure as well as add any additional comments. These could include changing the wording, adding a missing item, or changing the meaning of the item.

	Essential	Useful but not essential	Not necessary	Comment
Tearing a sheet of paper into many pieces	☐	☐	☐	☐
Cutting geometric shapes (circle, square, triangle)	☐	☐	☐	☐
Using scissors to snip the paper	☐	☐	☐	☐
Stringing beads together	☐	☐	☐	☐
Folding a piece of paper	☐	☐	☐	☐
Stacking blocks	☐	☐	☐	☐
Opening a band-aid	☐	☐	☐	☐

	Essential	Useful but not essential	Not necessary	Comment
Write your name or draw something	☐	☐	☐	☐
Opening a jar	☐	☐	☐	☐
Closing a jar	☐	☐	☐	☐
Turning over a card	☐	☐	☐	☐

Please provide any additional comments for the domain **"Fine Hand Manipulation"**

For the domain **"Throwing"** please rate the following items for inclusion in the SHAPE-UP measure as well as add any additional comments. These could include changing the wording, adding a missing item, or changing the meaning of the item.

	Essential	Useful but not essential	Not necessary	Comment
Throwing a ball overhead	☐	☐	☐	☐
Throwing a ball underhand	☐	☐	☐	☐

Please provide any additional comments for the domain **"Throwing"**

For the domain **"Catching"** please rate the following items for inclusion in the SHAPE-UP measure as well as add any additional comments. These could include changing the wording, adding a missing item, or changing the meaning of the item.

	Essential	Useful but not essential	Not necessary	Comment
Catching a ball overhead one handed	☐	☐	☐	☐
Catching a ball underhand one handed	☐	☐	☐	☐
Catching a ball overhead two handed	☐	☐	☐	☐
Catching a ball underhand two handed	☐	☐	☐	☐

Please provide any additional comments for the domain **"Catching"**

For the domain **"Dressing"** please rate the following items for inclusion in the SHAPE-UP measure as well as add any additional comments. These could include changing the wording, adding a missing item, or changing the meaning of the item.

	Essential	Useful but not essential	Not necessary	Comment
Puts on clothes over-head (shirt, sweater, hat)	☐	☐	☐	☐
Puts on open shirt	☐	☐	☐	☐
Removes shirt	☐	☐	☐	☐
Buttoning	☐	☐	☐	☐

	Essential	Useful but not essential	Not necessary	Comment
Unbuttoning	☐	☐	☐	☐
Starts a zipper	☐	☐	☐	☐
Pull up zipper	☐	☐	☐	☐
Pulls down zipper	☐	☐	☐	☐
Snaps fasteners	☐	☐	☐	☐
Unsnaps fasteners	☐	☐	☐	☐
Pulls on pants	☐	☐	☐	☐
Pulls down (remove) pants	☐	☐	☐	☐
Puts on socks	☐	☐	☐	☐
Remove socks	☐	☐	☐	☐
Puts on shoes	☐	☐	☐	☐
Ties shoelaces	☐	☐	☐	☐
Removes shoes	☐	☐	☐	☐

Please provide any additional comments for the domain **"Dressing"**

For the domain **"Feeding"** please rate the following items for inclusion in the SHAPE-UP measure as well as add any additional comments. These could include changing the wording, adding a missing item, or changing the meaning of the item.

	Essential	Useful but not essential	Not necessary	Comment
Reaches mouth	☐	☐	☐	☐
Cuts food using knife	☐	☐	☐	☐
Picks up food using fork	☐	☐	☐	☐

Please provide any additional comments for the domain **"Feeding"**

For the domain **"Toileting"** please rate the following items for inclusion in the SHAPE-UP measure as well as add any additional comments. These could include changing the wording, adding a missing item, or changing the meaning of the item.

	Essential	Useful but not essential	Not necessary	Comment
Places sticker on lower back (proxy for reaching to wipe buttocks)	☐	☐	☐	☐

Please provide any additional comments for the domain **"Toileting"**

Additional Comments

Please add any comments you may have regarding any additional items you feel should be included in the SHAPE-UP measure that were not included in the survey.

Thank you for your participation! We will collate all the responses and send out the items with highest level of agreement and proposed scoring scheme for a second survey.
If you have any questions regarding the survey or the research project, please contact the clinical research coordinator Kathleen Montpetit at kmontpetit@shrinenet.org
