# Supplementary material for: Stakeholder engagement in the development of an upper extremity outcome measure for children with rare musculoskeletal conditions
Source: Res Involv Engagem. 2023 Aug 8;9:64. doi: 10.1186/s40900-023-00479-6 (PMC10408044; doi:10.1186/s40900-023-00479-6)
Supplement: Supplementary file 2 — Additional file 2. Second-round survey questionnaire. [file 40900_2023_479_MOESM2_ESM.pdf]

The purpose of the SHAPE-UP is to assess upper extremity (UE) function in children with AMC with upper limb involvement. The SHAPE-UP will describe the impairments, activity limitations, and participation restrictions in the performance of daily tasks in children with AMC to guide treatment decision-making and evaluation treatment effectiveness for the UE.

In the tasks provided below, this will be the scoring guide implemented for the SHAPE-UP:

0 = Unable - The child is unable to complete any component of the task.
1 = **Partial** completion of task **passively** - The child can partially complete the task using passive range of motion.
2 = **Partial** completion of task **actively** - The child can partially compelte the task using active range of motion.
3 = **Completion** of task **passively** - The child can complete the task using passively range of motion.
4 = **Completion** of task **actively** - The child can complete the task using active range of motion.

Block 0 - Introduction

Thank you for participating in the SHAPE-UP Delphi Round #2. You are being invited to participate as you have previously completed round 1 of the survey. We have since taken into account all comments and feedback and have created a first version of the SHAPE-UP measure that we will present shortly.

Please note there are 12 tasks in the SHAPE-UP measure as well as 3 descriptive questions. The purpose of the measure as well as the scoring grid will be provided throughout the survey.

Please download the following PDF as a reference for the scoring tables if you require them.
LINK

We appreciate your time and efforts in completing this survey.

Block 1

Do you have any comments regarding the following task:

Task #1: Grasp/pick up a cheerio, bring it to your mouth, place it back down in front of you, and release it/let it go.

Grasp/pick up a cheerio	
bring it to your mouth	
place it back down in front of you	
release it/let it go	

Which word do you prefer?

- ○ Grasp a cheerio
- ○ Pick up a cheerio

Which word do you prefer?

- ○ release it
- ○ let it go

For the following task, we would like you to comment if an additional joint should be analyzed. We believe this task is focused more on the ***Thumb, Fingers, Elbow***.

Task #1: ${q://QID9/ChoiceGroup/SelectedChoices} a cheerio, bring it to your mouth, place it back down in front of you, and ${q://QID17/ChoiceGroup/SelectedChoices}.

Item	Score					Asst. Device	Arm		Analysis of Joint Motion and Position					
							R	L	Thumb	Fingers	Wrist	Forearm	Elbow	Shoulder
Grasp/ Pick up	0	1	2	3	4				Palm/close/open	Flexion/neutral/extension	Flexion/neutral/extension	-	-	-
Bring to mouth	0	1	2	3	4				-	-	-	Pronated/ /neutral/supination	Extension/neutral/flexion	-
Bring back to table	0	1	2	3	4				-	-	-	Supination/neutral/ pronation	Flexion/neutral/extension	-
Release/let go	0	1	2	3	4				Palm/close/open	Flexion/neutral/extension	Flexion/neutral/extension	-	-	-

Remember that for the Analysis of Joint Motion and Position, the scoring options represent less to more function/optimal position for the item in question.

Please select which joint(s) should be **targeted** in the scoring sheet for the specified task.

	Thumb Score		Fingers Score		Wrist Score		Forearm Score		Elbow Score		Shoulder Score	
	Yes	No	Yes	No	Yes	No	Yes	No	Yes	No	Yes	No

	Thumb Score		Fingers Score		Wrist Score		Forearm Score		Elbow Score		Shoulder Score	
	Yes	No	Yes	No	Yes	No	Yes	No	Yes	No	Yes	No
Grasp/ Pick up	O	O	O	O	O	O	O	O	O	O	O	O
Bring to mouth	O	O	O	O	O	O	O	O	O	O	O	O
Bring back to table	O	O	O	O	O	O	O	O	O	O	O	O
Release/ let go	O	O	O	O	O	O	O	O	O	O	O	O

Other thoughts or notes regarding Task #1: ${q://QID9/ChoiceGroup/SelectedChoices} a cheerio, bring it to your mouth, place it back down in front of you, and ${q://QID17/ChoiceGroup/SelectedChoices}.

Block 2

Do you have any comments regarding the following task:

Task #2: Grasp/pick up a water bottle/can, bring it to your mouth, place it back down in front of you, and release it/let it go.

Grasp/pick up a water bottle/can

bring it to your mouth

place it back down in front of you

release it/let it go

Which word do you prefer?

- O Grasp
- O Pick up

Which word do you prefer?

- ○ water bottle
- ○ can

Which word do you prefer?

- ○ release it
- ○ let it go

For the following task, we would like you to comment if an additional joint should be analyzed. We believe this task is focused more on the ***Thumb, Fingers, Elbow***.

Task #2: ${q://QID13/ChoiceGroup/SelectedChoices}
a ${q://QID18/ChoiceGroup/SelectedChoices}, bring it to your mouth, place it back down in front of you, and ${q://QID19/ChoiceGroup/SelectedChoices}.

Item	Score					Asst. Device	Arm		Analysis of Joint Motion and Position					
							R	L	Thumb	Fingers	Wrist	Forearm	Elbow	Shoulder
Grasp/ Pick up	0	1	2	3	4				Palm/close/open	Flexion/neutral/extension	Flexion/neutral/extension	-	-	-
Bring to mouth	0	1	2	3	4				-	-	-	Pronated/ /neutral/supination	Extension/neutral/flexion	-
Bring back to table	0	1	2	3	4				-	-	-	Supination/neutral/ pronation	Flexion/neutral/extension	-
Release/let go	0	1	2	3	4				Palm/close/open	Flexion/neutral/extension	Flexion/neutral/extension	-	-	-

Remember that for the Analysis of Joint Motion and Position, the scoring options represent less to more function/optimal position for the item in question.

Please select which joint(s) should be **targeted** in the scoring sheet for the specified task.

	Thumb Score		Fingers Score		Wrist Score		Forearm Score		Elbow Score		Shoulder Score	
	Yes	No	Yes	No	Yes	No	Yes	No	Yes	No	Yes	No
Grasp/ Pick up	○	○	○	○	○	○	○	○	○	○	○	○
Bring to mouth	○	○	○	○	○	○	○	○	○	○	○	○
Bring back to table	○	○	○	○	○	○	○	○	○	○	○	○
Release/ let go	○	○	○	○	○	○	○	○	○	○	○	○

Other thoughts or notes regarding Task #2: ${q://QID13/ChoiceGroup/SelectedChoices}
a ${q://QID18/ChoiceGroup/SelectedChoices}, bring it to your mouth, place it back down in front of you, and ${q://QID19/ChoiceGroup/SelectedChoices}.

Block 3

Do you have any comments regarding the following task:

Task #3: Open the jar, pour out a few beads/macaroni/buttons, string 2 or 3 together, and close the jar [small size jar e.g. spice jar or salt/paper shaker]

Open the jar

Pour out a few beads/macaroni/buttons

String 2 or 3 together

Close the jar

Which word do you prefer?

- beads
- macaroni
- buttons

For the following task, we would like you to comment if an additional joint should be analyzed. We believe this task is focused more on the ***Thumb, Fingers, Wrist***.

Task #3: Open the jar, pour out a few ${q://QID21/ChoiceGroup/SelectedChoices}, string 2 or 3 together, and close the jar.

Item	Score					Asst. Device	Arm		Analysis of Joint Motion and Position					
							R	L	Thumb	Fingers	Wrist	Forearm	Elbow	Shoulder
Stabilizes jar	0	1	2	3	4				Palm/close/open	No finger movement/partial finger movement/ full finger movement	Extension/ flexion/neutral	Supination/pronation/ neutral	-	-
Open jar	0	1	2	3	4				Palm/close/open	No finger movement/partial finger movement/ full finger movement	Extension/ Neutral/ flexion	Supination/neutral/ pronation	-	-
Pour out	0	1	2	3	4				Palm/close/open	-	-	Supination/neutral/ pronation	-	-
Hold small item	0	1	2	3	4				Palm/close/open	Fist/Finger adduction/True opposition	-	-	-	-
String	0	1	2	3	4				Palm/close/open	Fist/Finger adduction/True opposition	-	-	-	-
Close jar	0	1	2	3	4				Palm/close/open	No finger movement/partial finger movement/ full finger movement	Extension/ Neutral/ flexion	Supination/neutral/ pronation	-	-

Remember that for the Analysis of Joint Motion and Position, the scoring options represent less to more function/optimal position for the item in question.

Please select which joint(s) should be **targeted** in the scoring sheet for the specified task.

	Thumb Score		Fingers Score		Wrist Score		Forearm Score		Elbow Score		Shoulder Score	
	Yes	No	Yes	No	Yes	No	Yes	No	Yes	No	Yes	No
Stabilizes jar	O	O	O	O	O	O	O	O	O	O	O	O
Open jar	O	O	O	O	O	O	O	O	O	O	O	O
Pour out	O	O	O	O	O	O	O	O	O	O	O	O
Hold small item	O	O	O	O	O	O	O	O	O	O	O	O
String	O	O	O	O	O	O	O	O	O	O	O	O
Close jar	O	O	O	O	O	O	O	O	O	O	O	O

Other thoughts or notes regarding Task #3: Open the jar, pour out a few ${q://QID21/ChoiceGroup/SelectedChoices}, string 2 or 3 together, and close the jar.

Block 4

Do you have any comments regarding the following task:

Task #4: Pick up the crayon/marker, write your name on this piece of paper, fold the paper, and cut it using the scissors. [Therapist to place crayon/marker and scissors across the midline]

Pick up the crayon/marker

Write your name on this piece of paper

Fold the paper

Cut it using the scissors

Which word do you prefer?

- ◯ crayon
- ◯ marker

For the following task, we would like you to comment if an additional joint should be analyzed. We believe this task is focused more on the ***Thumb, Fingers, Wrist***.

Task #4: Pick up the ${q://QID28/ChoiceGroup/SelectedChoices}, write your name on this piece of paper, fold the paper, and cut it using the scissors

Item	Score					Asst. Device	Arm		Analysis of Joint Motion and Position					
							R	L	Thumb	Fingers	Wrist	Forearm	Elbow	Shoulder
pick up	0	1	2	3	4				Palm/close/open	No finger movement/partial finger movement/ full finger movement	Flexion/neutral/ extension	supination/neutral/pronation	-	-
write name	0	1	2	3	4				Palm/close/open	No finger movement/partial finger movement/ full finger movement	Flexion/neutral/ extension	supination/neutral/pronation	-	-
fold paper	0	1	2	3	4				Palm/close/open	No finger movement/partial finger movement/ full finger movement	Flexion/neutral/ extension	-	-	-
cut	0	1	2	3	4				Palm/close/open	No finger movement/partial finger movement/ full finger movement	Flexion/neutral/ extension	supination/pronation/neutral	-	-
stabilize paper to cut	0	1	2	3	4				Palm/close/open	No finger movement/partial finger movement/ full finger movement	Flexion/neutral/ extension	supination/pronation/neutral	-	-

Remember that for the Analysis of Joint Motion and Position, the scoring options represent less to more function/optimal position for the item in question.

Please select which joint(s) should be **targeted** in the scoring sheet for the specified task.

	Thumb Score		Fingers Score		Wrist Score		Forearm Score		Elbow Score		Shoulder Score	
	Yes	No	Yes	No	Yes	No	Yes	No	Yes	No	Yes	No
Pick up	◯	◯	◯	◯	◯	◯	◯	◯	◯	◯	◯	◯
Write name	◯	◯	◯	◯	◯	◯	◯	◯	◯	◯	◯	◯
Fold paper	◯	◯	◯	◯	◯	◯	◯	◯	◯	◯	◯	◯
Cut	◯	◯	◯	◯	◯	◯	◯	◯	◯	◯	◯	◯
Stabilize paper to cut	◯	◯	◯	◯	◯	◯	◯	◯	◯	◯	◯	◯

Other thoughts or notes regarding Task #4: Pick up the ${q://QID28/ChoiceGroup/SelectedChoices}, write your name on this piece of paper, fold the paper, and cut it using the scissors.

Block 5

Do you have any comments regarding the following task:

Task #5: Pick up the Play-Doh using the fork and bring it to your mouth. [Therapist to place items in front of child]

Pick up the Play-Doh using the fork

Bring it to your mouth

For the following task, we would like you to comment if an additional joint should be analyzed. We believe this task is focused more on the ***Wrist and Forearm***.

Task #5: Pick up the Play-Doh using the fork and bring it to your mouth.

Item	Score					Asst. Device	Arm		Analysis of Joint Motion and Position					
							R	L	Thumb	Fingers	Wrist	Forearm	Elbow	Shoulder
Pick up the play doh	0	1	2	3	4				palm/close/open	Fist /Finger adduction/True opposition	flexion/extension/neutral	supination/pronation/neutral	-	-
bring fork to mouth	0	1	2	3	4				-	-	-	Pronation//neutral/supination	Extension/neutral/flexion	-

Remember that for the Analysis of Joint Motion and Position, the scoring options represent less to more function/optimal position for the item in question.

Please select which joint(s) should be **targeted** in the scoring sheet for the specified task.

	Thumb Score		Fingers Score		Wrist Score		Forearm Score		Elbow Score		Shoulder Score	
	Yes	No	Yes	No	Yes	No	Yes	No	Yes	No	Yes	No
Pick up the Play-Doh	○	○	○	○	○	○	○	○	○	○	○	○
Bring fork to mouth	○	○	○	○	○	○	○	○	○	○	○	○

Other thoughts or notes regarding Task #5: Pick up the Play-Doh using the fork and bring it to your mouth.

Block 6

Do you have any comments regarding the following task:

Task #6: Reach for a small-size (e.g., tennis ball) ball placed on the floor, throw the ball underhand. Repeat task, throwing ball overhead.

Reach for small-size ball placed on the floor

throw the ball underhand

Repeat task

Throwing ball overhead

For the following task, we would like you to comment if an additional joint should be analyzed. We believe this task is focused more on the ***Elbow and Shoulder***.

Task #6: Reach for a small-size (e.g., tennis ball) ball placed on the floor, throw the ball underhand. Repeat task, throwing ball overhead.

Item	Score					Asst. Device	Arm		Analysis of Joint Motion and Position					
							R	L	Thumb	Fingers	Wrist	Forearm	Elbow	Shoulder
Reach ball on floor	0	1	2	3	4				-	-	Flexion/neutral/ extension	Supination/ neutral/pronation	Flexion/neutral /extension	Extension/neutral/ flexion
throw ball underhand (winding/extend back at shoulder)	0	1	2	3	4				-	-	Flexion/neutral/ extension	Supination/ neutral/pronation	Flexion/neutral /extension	Flexion/neutral/ extension
throw ball underhand (release at shoulder)	0	1	2	3	4						Flexion/neutral/ extension	Supination/ neutral/pronation	Flexion/neutral /extension	Extension/neutral/ flexion
throw ball overhand	0	1	2	3	4				-	-	Flexion/neutral/ extension	Pronation/neutral/ supination	Flexion/neutral /extension	Extension/neutral/ flexion

Remember that for the Analysis of Joint Motion and Position, the scoring options represent less to more function/optimal position for the item in question.

Please select which joint(s) should be **targeted** in the scoring sheet for the specified task.

	Thumb Score		Fingers Score		Wrist Score		Forearm Score		Elbow Score		Shoulder Score	
	Yes	No	Yes	No	Yes	No	Yes	No	Yes	No	Yes	No
Reach ball on floor	○	○	○	○	○	○	○	○	○	○	○	○

	Thumb Score		Fingers Score		Wrist Score		Forearm Score		Elbow Score		Shoulder Score	
	Yes	No	Yes	No	Yes	No	Yes	No	Yes	No	Yes	No
Throw ball underhand (winding/extend at shoulder)	○	○	○	○	○	○	○	○	○	○	○	○
Throw ball underhand (release at shoulder)	○	○	○	○	○	○	○	○	○	○	○	○
Throw ball overhead	○	○	○	○	○	○	○	○	○	○	○	○

Other thoughts or notes regarding Task #6: Reach for a small-size (e.g., tennis ball) ball placed on the floor, throw the ball underhand. Repeat task, throwing ball overhead.

Block 7

Do you have any comments regarding the following task:

Task #7: Reach for a medium-sized (e.g., basket ball) ball placed on the floor, throw the ball underhand. Repeat task, throwing ball overhead.

Reach for medium-sized ball placed on the floor

throw the ball underhand

Repeat task

Throwing ball overhead

For the following task, we would like you to comment if an additional joint should be analyzed. We believe this task is focused more on the ***Elbow and Shoulder***.

Task #7: Reach for a medium-sized (e.g., tennis ball) ball placed on the floor, throw the ball underhand. Repeat task, throwing ball overhead.

Item	Score					Asst. Device	Arm		Analysis of Joint Motion and Position					
							R	L	Thumb	Fingers	Wrist	Forearm	Elbow	Shoulder
Reach ball on floor	0	1	2	3	4				-	-	Flexion/neutral/ extension	Supination/ neutral/pronation	Flexion/neutral /extension	Extension/neutral/ flexion
throw ball underhand (winding/extend back at shoulder)	0	1	2	3	4				-	-	Flexion/neutral/ extension	Supination/ neutral/pronation	Flexion/neutral /extension	Flexion/neutral/ extension
throw ball underhand (release at shoulder)	0	1	2	3	4						Flexion/neutral/ extension	Supination/ neutral/pronation	Flexion/neutral /extension	Extension/neutral/ flexion
throw ball overhand	0	1	2	3	4				-	-	Flexion/neutral/ extension	Pronation/neutral/ supination	Flexion/neutral /extension	Extension/neutral/ flexion

Remember that for the Analysis of Joint Motion and Position, the scoring options represent less to more function/optimal position for the item in question.

Please select which joint(s) should be **targeted** in the scoring sheet for the specified task.

	Thumb Score		Fingers Score		Wrist Score		Forearm Score		Elbow Score		Shoulder Score	
	Yes	No	Yes	No	Yes	No	Yes	No	Yes	No	Yes	No
Reach ball on floor	○	○	○	○	○	○	○	○	○	○	○	○
Throw ball underhand (winding/extend at shoulder)	○	○	○	○	○	○	○	○	○	○	○	○
Throw ball underhand (release at shoulder)	○	○	○	○	○	○	○	○	○	○	○	○
Throw ball overhead	○	○	○	○	○	○	○	○	○	○	○	○

Other thoughts or notes regarding Task #7: Reach for a medium-sized (e.g., basket ball) ball placed on the floor, throw the ball underhand. Repeat task, throwing ball overhead.

Block 8

Do you have any comments regarding the following task:

Task #8: Put on a T-shirt overhead and take off the T-shirt.

Put on a T-shirt overhead

Take off the T-shirt

For the following task, we would like you to comment if an additional joint should be analyzed. We believe this task is focused more on the ***Elbow and Shoulder***.

Task #8: Put on a T-shirt overhead and take off the T-shirt.

Item	Score					Asst. Device	Arm		Analysis of Joint Motion and Position					
							R	L	Thumb	Fingers	Wrist	Forearm	Elbow	Shoulder
put on shirt overhead	0	1	2	3	4				-	-	-	Pronated/supinated/neutral	Flexion/neutral/extension	no shoulder movement/partial shoulder movement/full shoulder movement
take off shirt	0	1	2	3	4				-	-	-	Pronated/supinated/neutral	Flexion/neutral/extension	no shoulder movement/partial shoulder movement/full shoulder movement

Remember that for the Analysis of Joint Motion and Position, the scoring options represent less to more function/optimal position for the item in question.

Please select which joint(s) should be **targeted** in the scoring sheet for the specified task.

	Thumb Score		Fingers Score		Wrist Score		Forearm Score		Elbow Score		Shoulder Score	
	Yes	No	Yes	No	Yes	No	Yes	No	Yes	No	Yes	No
Put on a T-shirt overhead	○	○	○	○	○	○	○	○	○	○	○	○
Take off the T-shirt	○	○	○	○	○	○	○	○	○	○	○	○

Other thoughts or notes regarding Task #8: Put on a T-shirt overhead and take off the T-shirt.

Block 9

Do you have any comments regarding the following task:

Task #9: Put on vest/sweater with zipper, fasten the zipper, pull it all the way up, and pull it back down. [Therapist to place zipper either at top or bottom of clothing depending on if the child can complete the task]

Put on vest/sweater with zipper

Fasten the zipper	
Pull it all the way up	
Pull it back down	

Which word do you prefer?

- vest
- sweater

For the following task, we would like you to comment if an additional joint should be analyzed. We believe this task is focused more on the ***Elbow and Shoulder***.

Task #9: Put on ${q://QID51/ChoiceGroup/SelectedChoices} with zipper, fasten the zipper, pull it all the way up, and pull it back down.

Item	Score					Asst. Device	Arm		Analysis of Joint Motion and Position					
							R	L	Thumb	Fingers	Wrist	Forearm	Elbow	Shoulder
put on	0	1	2	3	4				-	-	-	-	No elbow movement/partial elbow movement/ full elbow movement	No shoulder movement/partial shoulder movement/ full shoulder movement
stabilize zipper	0	1	2	3	4				palm/close/open	No finger movement/partial finger movement/ full finger movement	flexion/extension/ neutral	supination/pronation/neutral	flexion/extension/neutral	External rotation/internal rotation/neutral
Attach zipper	0	1	2	3	4				palm/close/open	No finger movement/partial finger movement/ full finger movement	flexion/extension/ neutral	supination/pronation/neutral	flexion/extension/neutral	External rotation/internal rotation/neutral
pull zipper up to top	0	1	2	3	4				palm/close/open	Extension/neutral/flexion	extension/flexion/ neutral	supination/pronation/neutral	extension/neutral/flexion	Internal rotation/External rotation/neutral
pull zipper all the way down	0	1	2	3	4				palm/close/open	Extension/neutral/flexion	extension/flexion/ neutral	supination/pronation/neutral	flexion/neutral/extension	Internal rotation/external rotation/neutral

Remember that for the Analysis of Joint Motion and Position, the scoring options represent less to more function/optimal position for the item in question.

Please select which joint(s) should be **targeted** in the scoring sheet for the specified task.

	Thumb Score		Fingers Score		Wrist Score		Forearm Score		Elbow Score		Shoulder Score	
	Yes	No	Yes	No	Yes	No	Yes	No	Yes	No	Yes	No
Put on	○	○	○	○	○	○	○	○	○	○	○	○
Stabilize the zipper	○	○	○	○	○	○	○	○	○	○	○	○
Attach the zipper	○	○	○	○	○	○	○	○	○	○	○	○
Pull the zipper all the way up	○	○	○	○	○	○	○	○	○	○	○	○

	Thumb Score		Fingers Score		Wrist Score		Forearm Score		Elbow Score		Shoulder Score	
	Yes	No	Yes	No	Yes	No	Yes	No	Yes	No	Yes	No
Pull the zipper down	○	○	○	○	○	○	○	○	○	○	○	○

Other thoughts or notes regarding Task #9: Put on ${q://QID51/ChoiceGroup/SelectedChoices} with zipper, fasten the zipper, pull it all the way up, and pull it back down. [Therapist to place zipper either at top or bottom of clothing depending on if the child can complete the task]

Block 10

Do you have any comments regarding the following task:

Task #10: Pull down your pants, reach bum/buttocks area, place a sticker on bum/buttocks area [proxy for wiping after bowel movement], place sticker in between legs [proxy for wiping after urination], and pull pants back up. [Therapist to provide loose fitting pants].

Pull down your pants

Reach bum/buttocks area

Place a sticker on bum/buttocks area

Place sticker in between legs

Pull pants back up

Which word do you prefer?

○ bum

○ buttock

For the following task, we would like you to comment if an additional joint should be analyzed. We believe this task is focused more on the ***Thumb, Wrist, Elbow***.

Task #10: Pull down your pants, reach ${q://QID56/ChoiceGroup/SelectedChoices} area, place a sticker on ${q://QID56/ChoiceGroup/SelectedChoices} area, place sticker in between legs, and pull pants back up.

Item	Score					Asst. Device	Arm		Analysis of Joint Motion and Position					
							R	L	Thumb	Fingers	Wrist	Forearm	Elbow	Shoulder
Pull down	0	1	2	3	4				palm/close/open	-	flexion/neutral/extension	-	no elbow movement/partial elbow movement/full elbow movement	-
Reach bum	0	1	2	3	4				-	-	-	-	flexion/neutral/extension	no shoulder movement/partial shoulder movement/full shoulder movement
Place sticker on bum	0	1	2	3	4				palm/close/open	no finger movement/partial finger movement/full finger movement	extension/neutral/flexion	pronation/neutral/supination	-	-
Place sticker between legs	0	1	2	3	4				palm/close/open	no finger movement/partial finger movement/full finger movement	extension/neutral/flexion	supination/neutral/pronation	-	-
Pull up pants	0	1	2	3	4				palm/close/open	no finger movement/partial finger movement/full finger movement	-	-	no elbow movement/partial elbow movement/full elbow movement	-

Remember that for the Analysis of Joint Motion and Position, the scoring options represent less to more function/optimal position for the item in question.

Please select which joint(s) should be **targeted** in the scoring sheet for the specified task.

	Thumb Score		Fingers Score		Wrist Score		Forearm Score		Elbow Score		Shoulder Score	
	Yes	No	Yes	No	Yes	No	Yes	No	Yes	No	Yes	No
Pull down	○	○	○	○	○	○	○	○	○	○	○	○
Reach bum	○	○	○	○	○	○	○	○	○	○	○	○
Place sticker on bum	○	○	○	○	○	○	○	○	○	○	○	○
Place sticker in between legs	○	○	○	○	○	○	○	○	○	○	○	○
Pull up pants	○	○	○	○	○	○	○	○	○	○	○	○

Other thoughts or notes regarding Task #10: Pull down your pants, reach ${q://QID56/ChoiceGroup/SelectedChoices} area, place a sticker on ${q://QID56/ChoiceGroup/SelectedChoices} area, place sticker in between legs, and pull pants back up.

Block 11

Do you have any comments regarding the following task:

Task #11: Put on a sock and take it off. [Note: Do not score this task if the child has a lower extremity contracture that prohibits task completion].

Put on a sock

Take it off

For the following task, we would like you to comment if an additional joint should be analyzed. We believe this task is focused more on the ***Thumb and Elbow***.

Task #11: Put on a sock and take it off.

Item	Score					Asst. Device	Arm		Analysis of Joint Motion and Position					
							R	L	Thumb	Fingers	Wrist	Forearm	Elbow	Shoulder
Put on sock over toe	0	1	2	3	4				palm/close/open	Extension/neutral/flexion	-	-	Flexion/neutral/extension	-
Pull sock over heel	0	1	2	3	4				palm/close/open	Extension/neutral/flexion	-	-	extension/neutral/flexion	-

Remember that for the Analysis of Joint Motion and Position, the scoring options represent less to more function/optimal position for the item in question.

Please select which joint(s) should be **targeted** in the scoring sheet for the specified task.

	Thumb Score		Fingers Score		Wrist Score		Forearm Score		Elbow Score		Shoulder Score	
	Yes	No	Yes	No	Yes	No	Yes	No	Yes	No	Yes	No
Put on sock over toe	O	O	O	O	O	O	O	O	O	O	O	O
Pull sock over heel	O	O	O	O	O	O	O	O	O	O	O	O

Other thoughts or notes regarding Task #11: Put on a sock and take it off. [Note: Do not score this task if the child has a lower extremity contracture that prohibits task

completion].

Block 12

Do you have any comments regarding the following task:

Task #12: Show us how you move from lying down on your back to a sitting position

Lying to sitting

For the following task, we would like you to comment if an additional joint should be analyzed. We believe this task is focused more on the ***Wrist, Forearm, Elbow***.

Task #12: Show us how you move from lying down on your back to a sitting position

Item	Score					Asst. Device	Arm		Analysis of Joint Motion and Position					
							R	L	Thumb	Fingers	Wrist	Forearm	Elbow	Shoulder
Lying to sitting	0	1	2	3	4				-	-	flexion/neutral/extension	supination/neutral/pronation	flexion/neutral/extension	-

Remember that for the Analysis of Joint Motion and Position, the scoring options represent less to more function/optimal position for the item in question.

Please select which joint(s) should be **targeted** in the scoring sheet for the specified task.

	Thumb Score		Fingers Score		Wrist Score		Forearm Score		Elbow Score		Shoulder Score	
	Yes	No	Yes	No	Yes	No	Yes	No	Yes	No	Yes	No
Lying to sitting	O	O	O	O	O	O	O	O	O	O	O	O

Other thoughts or notes regarding Task #12: Show us how you move from lying down on your back to a sitting position

Block 13 - Qualitative Questions

In addition to the quantitative tasks and scoring above, we'd like to ask a few descriptive questions.
Please provide comments and feedback on the next 3 qualitative questions.

Does the child use their arms for:

- Using a mobility device ☐ Yes ☐ No. If yes, describe:

____

____

____

Does the child use their arms for:

- Shifting /changing/moving your body (e.g., getting on or off couch/toilet/etc.) ☐ Yes ☐ No. If yes, describe:

____

____

____

Are you using a splint for the tasks included: ☐ Yes ☐ No; ☐ Right ☐ Left. If yes, describe:

____

____

____

Block 14 - Overall Comments

Thank you for completing the survey. Please provide any feedback regarding the layout of the scoring sheet, the overall content of the SHAPE-UP, if any items/tasks are missing, or any other comments.

Powered by Qualtrics
