# Supplementary material for: Stakeholder engagement in the development of an upper extremity outcome measure for children with rare musculoskeletal conditions
Source: Res Involv Engagem. 2023 Aug 8;9:64. doi: 10.1186/s40900-023-00479-6 (PMC10408044; doi:10.1186/s40900-023-00479-6)
Supplement: Supplementary file 3 — Additional file 3. Third-round survey questionnaire. [file 40900_2023_479_MOESM3_ESM.pdf]

Block 0 - Introduction

Thank you for participating in the third and final round of the SHAPE-UP Delphi. We have taken into account all your comments and feedback and have modified the SHAPE-UP outcome measure. The yellow highlights indicate all the modifications and edits made to the SHAPE-UP between Round 2 and Round 3.

Please note there are now 11 tasks as well as 3 descriptive questions. Each task is further broken down into a series of items and scored accordingly. Some tasks are unilateral, others bilateral, but this has been accounted for in the scoring table.

As a reminder, the purpose of the SHAPE-UP is to assess upper extremity (UE) function in children with AMC with upper limb involvement. The SHAPE-UP will describe the impairments, activity limitations, and participation restrictions in the performance of daily tasks in children with AMC to guide treatment decision-making and evaluation treatment effectiveness for the UE.

The scoring guide below describes how each item within the 11 tasks will be scored.

**Task Completion**
0 = **unable** - The child is unable to complete any component of the task.
1 = **partial** completion of task **passively** - The child can partially complete the task using passive range of motion.
2 = **partial** completion of task **actively** - The child can partially complete the task using active range of motion.

3 = **completion** of task **passively** - The child can complete the task using passive range of motion.
4 = **completion** of task **actively** - The child can complete the task using active range of motion.
**Analysis of Joint Motion and Position**
Each joint of the UE (**Thumb, Fingers, Wrist, Forearm, Elbow, Shoulder**) is labeled and categorized from **least to most optimal position** for the specific item being assessed. If the item scoring option is "-", the focus of the task and item is not on that specific joint.

In addition, we strongly recommend you download the following PDF as a reference guide for the scoring tables for all 11 tasks and 3 descriptive questions. This will allow you to get a complete picture of the SHAPE-UP in it's entirety.

For this final round Delphi, we ask that you look at the SHAPE-UP as a whole and provide any comments regarding scoring, overall layout, and clarity of content.

We appreciate your time and efforts in completing this survey.

Block 1

Task #1. Pick up a cheerio, bring it to your mouth, place it back down in front of you, and let it go.

Item	Task completion					Arm		Analysis of Joint Motion and Position					
						R	L	Thumb	Fingers	Wrist	Forearm	Elbow	Shoulder
Pick up cheerio	0	1	2	3	4			Palm/Close/Open	Flexion/Neutral/Extension	Flexion/Neutral/Extension	-	-	-
Bring it to mouth	0	1	2	3	4			-	-	-	Pronated/ /Neutral/Supination	Extension/Neutral/Flexion	No External Rotation/Partial External Rotation/Full External Rotation
Place it back down in front of you	0	1	2	3	4			-	-	-	Supination/Neutral/ Pronation	Flexion/Neutral/Extension	-
Let it go	0	1	2	3	4			Palm/Close/Open	Flexion/Neutral/Extension	Flexion/Neutral/Extension	-	-	-

Remember that for the Analysis of Joint Motion and Position, the scoring options indicate less to more function/optimal position for the specific item within the task.

Additional comments regarding Task #1: Pick up a cheerio, bring it to your mouth, place it back down in front of you, and let it go.

Block 2

Task #2: Grasp a water bottle, bring it to your mouth, place it back down in front of you, and let it go.

Item	Score					Arm		Analysis of Joint Motion and Position					
						R	L	Thumb	Fingers	Wrist	Forearm	Elbow	Shoulder
Grasp a water bottle	0	1	2	3	4			Palm/Close/Open	Flexion/Neutral/Extension	Flexion/Neutral/Extension	Supination/Neutral/Pronation	-	-
Bring it to mouth	0	1	2	3	4			-	-	Flexion/Neutral/Extension	Pronation/ /Neutral/Supination	Extension/Neutral/Flexion	No External Rotation/Partial External Rotation/Full External Rotation
Place it back down in front of you	0	1	2	3	4			-	-	Flexion/Neutral/Extension	Supination/Neutral/ Pronation	Flexion/Neutral/Extension	-
Let it go	0	1	2	3	4			Palm/Close/Open	Flexion/Neutral/Extension	Flexion/Neutral/Extension	Supination/Neutral/Pronation	-	-

Remember that for the Analysis of Joint Motion and Position, the scoring

options indicate less to more function/optimal position for the specific item within the task.

Additional comments regarding Task #2. Grasp a water bottle, bring it to your mouth, place it back down in front of you, and let it go.

Block 3

Task #3. Open the jar, pour out a few cubes, string 3 together, and close the jar.

Item	Score					Arm		Analysis of Joint Motion and Position					
						R	L	Thumb	Fingers	Wrist	Forearm	Elbow	Shoulder
Stabilizes jar	0	1	2	3	4			Palm/Close/Open	No Finger Movement/Partial Finger Movement/ Full Finger Movement	Extension/ Flexion/Neutral	Supination/Pronation/ Neutral	-	-
Open jar	0	1	2	3	4			Palm/Close/Open	No Finger Movement/Partial Finger Movement/ Full Finger Movement	Extension/ Neutral/ Flexion	Supination/Neutral/ Pronation	-	-
Pour out	0	1	2	3	4			Palm/Close/Open	No Finger Movement/Partial Finger Movement/ Full Finger Movement	-	Supination/Neutral/ Pronation	-	-
Hold bead	0	1	2	3	4			Palm/Close/Open	Fingers flexed (hand fisted) /Hand opened (not fisted)/Grasp between thumb and fingers (opposition)	-	-	-	-
String 3	0	1	2	3	4			Palm/Close/Open	Fingers flexed (hand fisted) /Hand opened (not fisted)/Grasp between thumb and fingers (opposition)	-	-	-	-
Close jar	0	1	2	3	4			Palm/Close/Open	No Finger Movement/Partial Finger Movement/ Full Finger Movement	Extension/ Neutral/ Flexion	Supination/Neutral/ Pronation	-	-

Remember that for the Analysis of Joint Motion and Position, the scoring options indicate less to more function/optimal position for the specific item within the task.

Additional comments regarding Task #3: Open the jar, pour out a few cubes, string 3 together, and close the jar.

Block 4

Task #4. Pick up the marker, write your name on this piece of paper with marker, fold the paper, and cut it using the scissors.

[Therapist to place uncapped marker across the midline]

Item	Score					Arm		Analysis of Joint Motion and Position					
						R	L	Thumb	Fingers	Wrist	Forearm	Elbow	Shoulder
Pick up the marker	0	1	2	3	4			Palm/Close/Open	No Finger Movement/Partial Finger Movement/ Full Finger Movement	Flexion/Neutral/ Extension	Supination/Neutral/Pronation	-	-
Write name	0	1	2	3	4			Palm/Close/Open	No Finger Movement/Partial Finger Movement/ Full Finger Movement	Flexion/Neutral/ Extension	Supination/Neutral/Pronation	-	-
Fold paper	0	1	2	3	4			Palm/Close/Open	No Finger Movement/Partial Finger Movement/ Full Finger Movement	Flexion/Neutral/ Extension	Supination/Neutral/Pronation	-	-
Cut paper	0	1	2	3	4			Palm/Close/Open	No Finger Movement/Partial Finger Movement/ Full Finger Movement	Flexion/Neutral/ Extension	Supination/Pronation/Neutral	-	-
Stabilize paper to cut	0	1	2	3	4			Palm/Close/Open	No Finger Movement/Partial Finger Movement/ Full Finger Movement	Flexion/Neutral/ Extension	Supination/Pronation/Neutral	-	-

Remember that for the Analysis of Joint Motion and Position, the scoring options indicate less to more function/optimal position for the specific item within the task.

Additional comments regarding Task #4. Pick up the marker, write your name on this piece of paper with marker, fold the paper, and cut it using the scissors.

Block 5

Task #5: Pick up the Play-Doh using the fork and bring to mouth.

[Therapist places piece of Play-Doh in front of child]

Item	Score					Arm		Analysis of Joint Motion and Position					
						R	L	Thumb	Fingers	Wrist	Forearm	Elbow	Shoulder
Pick Up the Play-Doh	0	1	2	3	4			Palm/Close/Open	Fingers flexed (hand fisted) /Hand opened (not fisted)/Grasp between thumb and fingers (opposition)	Flexion/Extension/Neutral	Supination/Pronation/ Neutral	-	No External Rotation/ Partial External Rotation/ Full External Rotation
Bring to Mouth	0	1	2	3	4			-	-	-	Pronation//Neutral/Supination	Extension/Neutral/Flexion	No External Rotation/ Partial External Rotation/ Full External Rotation

Remember that for the Analysis of Joint Motion and Position, the scoring options indicate less to more function/optimal position for the specific item within the task.

Additional comments regarding Task #5: Pick up the Play-Doh using the fork and bring to mouth.

Block 6

Task #6: Grasp a small-size ball (e.g., tennis ball), throw the ball underhand. Repeat task, throwing ball overhead.

Item	Score					Arm		Analysis of Joint Motion and Position					
						R	L	Thumb	Fingers	Wrist	Forearm	Elbow	Shoulder
Grasp small size ball	0	1	2	3	4			-	-	Flexion/Neutral/ Extension	Supination/ Neutral/Pronation	Flexion/Neutral /Extension	Extension/Neutral/ Flexion
Wind up in preparation to throw ball underhand	0	1	2	3	4			-	-	Flexion/Neutral/ Extension	Supination/ Neutral/Pronation	Flexion/Neutral /Extension	Flexion/Neutral/ Extension
Throw ball underhand	0	1	2	3	4			-	-	Flexion/Neutral/ Extension	Supination/ Neutral/Pronation	Flexion/Neutral /Extension	Extension/Neutral/ Flexion
Wind up in preparation to throw ball overhead	0	1	2	3	4			-	-	Flexion/Neutral/ Extension	Supination/ Neutral/Pronation	Flexion/Neutral /Extension	Flexion/Neutral/ Extension
Throw ball overhead								-	-	Flexion/Neutral/ Extension	Supination/ Neutral/Pronation	Flexion/Neutral /Extension	Extension/Neutral/ Flexion

Remember that for the Analysis of Joint Motion and Position, the scoring options indicate less to more function/optimal position for the specific item within the task.

Additional comments regarding Task #6: Grasp a small-size ball (e.g., tennis ball), throw the ball underhand. Repeat task, throwing ball overhead.

Block 7

Task #7: Grasp a medium-size ball (e.g., basket ball), throw the ball underhand. Repeat task, throwing ball overhead.

Underhand

Grasping bilaterally ☐ Yes ☐ No

Throwing bilaterally ☐ Yes ☐ No

Overhead

Grasping bilaterally ☐ Yes ☐ No

Throwing bilaterally ☐ Yes ☐ No

Item	Score					Arm		Analysis of Joint Motion and Position					
						R	L	Thumb	Fingers	Wrist	Forearm	Elbow	Shoulder
Grasp medium size ball	0	1	2	3	4			-	-	Flexion/Neutral/ Extension	Supination/ Neutral/Pronation	Flexion/Neutral /Extension	Extension/Neutral/ Flexion
Wind up in preparation to throw ball underhand	0	1	2	3	4			-	-	Flexion/Neutral/ Extension	Supination/ Neutral/Pronation	Flexion/Neutral /Extension	Flexion/Neutral/ Extension
Throw ball underhand	0	1	2	3	4			-	-	Flexion/Neutral/ Extension	Supination/ Neutral/Pronation	Flexion/Neutral /Extension	Extension/Neutral/ Flexion
Wind up in preparation to throw ball overhead								-	-	Flexion/Neutral/ Extension	Supination/ Neutral/Pronation	Flexion/Neutral /Extension	Extension/Neutral/ Flexion
Throw ball overhead	0	1	2	3	4			-	-	Flexion/Neutral/ Extension	Supination/Neutral/ Pronation	Flexion/Neutral /Extension	Extension/Neutral/ Flexion

Remember that for the Analysis of Joint Motion and Position, the scoring options indicate less to more function/optimal position for the specific item within the task.

Additional comments regarding Task #7: Grasp a medium-size ball (e.g., basket ball), throw the ball underhand. Repeat task, throwing ball overhead.

Block 8

Task #8: Put on and take off a T-shirt.

[Note that the following items do not need to be completed in the presented order.]

Item	Score					Arm		Analysis of Joint Motion and Position					
						R	L	Thumb	Fingers	Wrist	Forearm	Elbow	Shoulder
Put T-shirt over head	0	1	2	3	4			-	-	-	Pronated/Supinated/Neutral	Flexion/Neutral/Extension	No Shoulder Movement/Partial Shoulder Movement/Full Shoulder Movement
Put arms in sleeves	0	1	2	3	4			-	-	-	Pronated/ Neutral/Supinated	Flexion/Neutral/Extension	Internal rotation/neutral/External rotation
Pull down T-shirt	0	1	2	3	4			Palm/Close/Open	Fingers flexed (hand fisted) /Hand opened (not fisted)/Grasp between thumb and fingers (opposition)	Extension/Flexion/ Neutral	Pronated/Supinated/Neutral	Flexion/Neutral/Extension	No Shoulder Movement/Partial Shoulder Movement/Full Shoulder Movement
Remove arms from sleeves	0	1	2	3	4			-	-	-	Pronated/Supinated/Neutral	Flexion/Neutral/Extension	No Shoulder Movement/Partial Shoulder Movement/Full Shoulder Movement
Remove T-shirt	0	1	2	3	4			Palm/Close/Open	No finger movement/partial finger movement/full finger movement	Flexion/neutral/ extension	Pronated/Supinated/Neutral	Flexion/Neutral/Extension	Flexion/neutral/ extension/

Remember that for the Analysis of Joint Motion and Position, the scoring options indicate less to more function/optimal position for the specific item within the task.

Additional comments regarding Task #8: Put on and take off a T-shirt.

Block 9

Task #9: Put on zippered item on upper body, fasten the zipper, pull it all the way up, and pull it back down.

[Therapist to place zipper either at top or bottom depending on if the child can complete the task; if child cannot fasten the zipper, therapist to attach zipper for them.]

Item	Score					Arm		Analysis of Joint Motion and Position					
						R	L	Thumb	Fingers	Wrist	Forearm	Elbow	Shoulder
Put on item of clothing	0	1	2	3	4			-	-	-	-	No Elbow Movement/ Partial Elbow Movement/ Full Elbow Movement	No Shoulder Movement/ Partial Shoulder Movement/ Full Shoulder Movement
Stabilize zipper	0	1	2	3	4			Palm/Close/Open	No Finger Movement/ Partial Finger Movement/ Full Finger Movement	Flexion/ Extension/ Neutral	Supination/ Pronation/ Neutral	Flexion/Extension/Neutral	External Rotation/ Internal Rotation/ Neutral
Fasten zipper	0	1	2	3	4			Palm/Close/Open	No Finger Movement/ Partial Finger Movement/ Full Finger Movement	Flexion/ Extension/ Neutral	Supination/ Pronation/ Neutral	Flexion/Extension/Neutral	External Rotation/ Internal Rotation/ Neutral
Pull zipper all the way up	0	1	2	3	4			Palm/Close/Open	Extension/Neutral/Flexion	Extension/ Flexion/ Neutral	Supination/ Pronation/ Neutral	Extension/Neutral/Flexion	Internal Rotation/ External Rotation/ Neutral
Pull zipper all the way down	0	1	2	3	4			Palm/Close/Open	Extension/Neutral/Flexion	Extension/ Flexion/ Neutral	Supination/ Pronation/ Neutral	Flexion/Neutral/Extension	Internal Rotation/ External Rotation/ Neutral

Remember that for the Analysis of Joint Motion and Position, the scoring options indicate less to more function/optimal position for the specific item within the task.

Additional comments regarding Task #9: Put on zippered item on upper body, fasten the zipper, pull it all the way up, and pull it back down.

Block 10

Task #10: Pull down your pants, reach buttocks area from the back while holding 2 squares of toilet paper, and now reach between legs from the front while holding 2 squares of toilet paper, and pull pants back up.

[Therapist to provide loose fitting pants]

Item	Score					Arm		Analysis of Joint Motion and Position					
						R	L	Thumb	Fingers	Wrist	Forearm	Elbow	Shoulder
Pull down pants	0	1	2	3	4			Palm/Close/Open	-	Flexion/Neutral/ Extension	-	No Elbow Movement/ Partial Elbow Movement/ Full Elbow Movement	-
Reach buttocks area [back]	0	1	2	3	4			-	-	-	-	Flexion/Neutral/Extension	No Shoulder Movement/ Partial Shoulder Movement/ Full Shoulder Movement
Reach between legs [front]	0	1	2	3	4			Palm/Close/Open	No Finger Movement/ Partial Finger Movement/ Full Finger Movement	Extension/Neutral/ Flexion	Supination/Neutral/ Pronation	-	-
Pull up pants	0	1	2	3	4			Palm/Close/Open	No Finger Movement/ Partial Finger Movement/ Full Finger Movement	-	-	No Elbow Movement/ Partial Elbow Movement/ Full Elbow Movement	-

Remember that for the Analysis of Joint Motion and Position, the scoring options indicate less to more function/optimal position for the specific item within the task.

Additional comments regarding Task #10: Pull down your pants, reach buttocks area from the back while holding 2 squares of toilet paper, and now reach between legs from the front while holding 2 squares of toilet paper, and pull pants back up.

Block 11

Task #11: Put on a sock and take it off.
[If LE contracture prohibits the task, check ☐ NO SCORE]

Item	Score					Arm		Analysis of Joint Motion and Position					
						R	L	Thumb	Fingers	Wrist	Forearm	Elbow	Shoulder
Put on sock over toes	0	1	2	3	4			Palm/Close/Open	Extension/Neutral/Flexion	-	-	Flexion/Neutral/Extension	-
Pull sock over heel	0	1	2	3	4			Palm/Close/Open	Extension/Neutral/Flexion	-	-	Extension/Neutral/Flexion	-

Remember that for the Analysis of Joint Motion and Position, the scoring options indicate less to more function/optimal position for the specific item within the task.

Additional comments regarding Task #11: Put on a sock and take it off.

Block 12 - Descriptive Questions

Does the child use their arms to:

- Propel a wheelchair ☐ Yes ☐ No. If yes, describe: ____________________

- Use a walking aid (cane, crutch, walker) ☐ Yes ☐ No. If yes, describe: ____________________

-Other: ____________ ☐ Yes ☐ No. If yes, describe: ____________________

Any additional comments regarding Descriptive Question #1 above:

Block 13

Does the child use their arms to perform the following transfers:

Transfers	Uses their arms		Cannot use their arms	Cannot complete due to LE contracture	Describe
	Right	Left			
Bed positioning					
Lying to sitting					
Sitting to standing					
Toilet transfer					
Bathtub/shower transfer					
Getting in and out of a car					
Other:					
Other:					
Other:					

Any additional comments regarding Descriptive Question #2 above:

Block 14

Does the child use a splint/orthosis when performing the tasks included in the SHAPE-UP:

☐ Yes ☐ No. If yes indicate side: ☐ Right ☐ Left, and describe type of splint and its use: ____________________

____

____

____

____

____

____

____

Any additional comments regarding Descriptive Question #3 above:

Block 14 - Overall Comments

Please provide any overall feedback regarding the layout of the scoring sheet, the overall content of the SHAPE-UP, or any other comments.

Powered by Qualtrics
